# Long-term risk of dementia after acute respiratory failure requiring intensive care unit admission

**DOI:** 10.1371/journal.pone.0180914

**Published:** 2017-07-24

**Authors:** Chih-Cheng Lai, Chung-Han Ho, Chin-Ming Chen, Shyh-Ren Chiang, Chien-Ming Chao, Wei-Lun Liu, Yu-Chieh Lin, Jhi-Joung Wang, Kuo-Chen Cheng

**Affiliations:** 1 Department of Intensive Care Medicine, Chi Mei Medical Center, Liouying, Taiwan; 2 Departments of Medical Research, Chi Mei Medical Center, Tainan, Taiwan; 3 Department of Pharmacy, Chia Nan University of Pharmacy and Science, Tainan, Taiwan; 4 Departments of Intensive Care Medicine, Chi Mei Medical Center, Tainan, Taiwan; 5 Chia Nan University of Pharmacy & Science, Tainan, Taiwan; 6 Departments of Internal Medicine, Mei Medical Center, Tainan, Taiwan; 7 Departments of Family Medicine, Chi Mei Medical Center, Tainan, Taiwan; 8 Departments of Anesthesiology, Chi Mei Medical Center, Tainan, Taiwan; 9 Department of Safety, Health, and Environmental Engineering, Chung Hwa University of Medical Technology, Tainan, Taiwan; National Yang-Ming University, TAIWAN

## Abstract

This retrospective, population-based cohort study aims to investigate the long-term risk of newly diagnosed dementia in patients discharged for acute respiratory failure that required mechanical ventilation (MV) and intensive care unit (ICU) admission. From the Taiwan National Health Insurance Research Database, first-time ICU patients using MV between June 1, 1998, and December 31, 2012, were enrolled, and they were followed-up until the earliest onset of one of our two endpoints: a new diagnosis of dementia (primary endpoint), or the end of the study. A total of 18,033 patients were enrolled and thirteen hundred eighty-seven patients had been newly diagnosed with dementia (mean onset: 3.2 years post-discharge). Patients ≥ 85 years old had the highest risk (multivariate analysis). Males had a lower risk than did females in both models (HR: 0.81, 95% CI: 0.72–0.9 in model 1; HR: 0.80, 95% CI: 0.72–0.89 in model 2). ICU stays > 5 days, hospital stays > 14 days, and more ICU readmissions were associated with a higher risk of developing dementia. In conclusion, the long-term risks of a subsequent diagnosis of dementia for acute respiratory failure with MV patients who survive to discharge increase with age and are higher in women than in men. Additionally, the longer the ICU or hospital stay is, and the more ICU readmissions a patient has, are both significantly associated with developing dementia.

## Introduction

Because of the increasing incidence of critical illnesses that require admission to the intensive care unit (ICU) and to the decreasing mortality rates of critically ill patients [1–3], the number of survivors of critical illness is rapidly rising. Although these survivors can recover from their acute illness, they can develop several long-term sequelae, e.g., deterioration of physical functions, post-traumatic stress disorder, depression, and cognitive impairments [4–7]. Several investigations [5, 8–13] have reported significant associations between the development of cognitive impairments, including dementia, and ICU admission, especially in elderly (≥ 65 years old) patients. However, the characteristics of ICU patients are heterogeneous and complex. In addition to the association between age, genetic factors, stroke, dyslipidemima, hypertension, diabetes mellitus, cardiovascular factors, smoking, kidney dysfunction, sepsis, and dementia were ever reported in general population and some critical ill patients [8,9,14–18], and we wondered whether this close relationship between dementia and admission to the ICU could be applied to all ICU patients.

Acute respiratory failure (ARF) and acute respiratory distress syndrome (ARDS), both of which often require invasive mechanical ventilation (MV), are two of the most common diseases that lead to admission to the ICU. One study [5] reported that, (a) 1 year after being discharged from the hospital, 43 of 55 (78%) ARDS survivors had all or at least one of the following: impaired memory, attention, concentration, and slower mental processing speed; and (b) 54 of 74 (73%) had neurocognitive sequelae at hospital discharge, 30 of 66 (46%) 1 year post-discharge, and 29 of 62 (46%) 2 years post-discharge. However, investigation on the long-term risk of dementia after admission to the ICU and required MV is limited.

We hypothesized that there is a long-term risk of newly-diagnosed dementia in survivors of ARF who are admitted to the ICU, and that the risk may be related to age and other hospital factors. In this study, we investigated the long-term risk of newly diagnosed dementia in patients discharged after ARF that required admission to the ICU, and we determined the effects of age, gender, and hospital factors on that risk.

## Materials and methods

### Data source

This retrospective, population-based cohort study used Taiwan’s National Health Insurance Research Database (NHIRD). Taiwan’s National Health Insurance is a single-payer compulsory system, which currently enrolls more than 23 million of the country’s legal residents (> 99.7% of Taiwan’s population). The NHIRD provides detailed healthcare services information about the clinical visits for each insured beneficiary. It uses International Classification of Diseases, Ninth Revision, Clinical Modification (ICD-9-CM) diagnostic and procedure codes. We used the Longitudinal Health Insurance Database 2000 (LHID2000), which contains 1 million randomly selected NHI beneficiaries (about 4.34% of Taiwan’s population) from the year 2000 Registry of Beneficiaries of the NHIRD; it is representative of the demographic distribution of the Taiwanese population. The database is a longitudinal cohort that contains data related to outpatient and inpatient medical care, diagnoses, surgical procedures, and prescribed medications from 1996 to 2013. The study was approved by the Institutional Review Board (IRB) at Chi Mei Medical Center. The data used in this study are de-identified, and released to the public for research purposes; therefore, the IRB waived informed consent from the enrolled patients (IRB no: 10411-E01).

### Patient selection and definition

**Fig 1:**
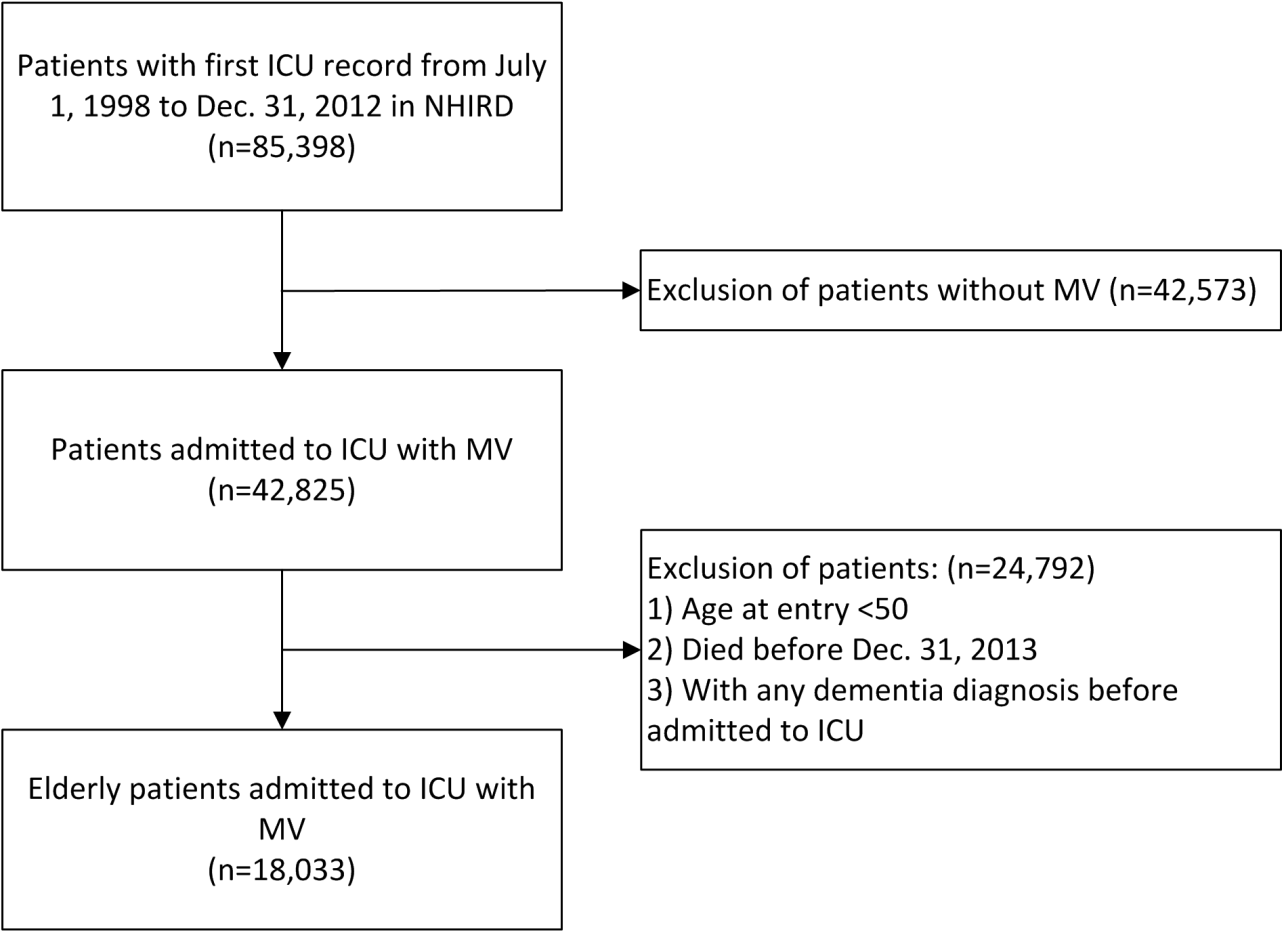
Flowchart of selection of study subjects.

Patients enrolled in this study had been admitted to the ICU and put on MV for the first time between June 1, 1998, and December 31, 2012. ICU admission and the use of invasive MV were defined according to medical expenditure applications. Patients who were < 50 years old, who had been admitted to the ICU but not put on MV, or who died during the follow up between June 1, 1998, and December 31, 2012 newly diagnosed dementia were excluded. Because we only investigated the long term risk of dementia, we excluded who had been diagnosed with dementia within 180 days after ICU admission. The start follow up point was therefore set as 180 days after the index ICU admission. We enrolled only new-onset dementia cases; therefore, prevalent cases of dementia before cohort entry were also excluded. Fig 1 presented the flowchart of selection of study subjects.

### Baseline variables

To be as comprehensive as possible in adjusting for factors that might affect outcome, we evaluated the following factors as potential confounders: gender, age, and possible risk factors for dementia—stroke (ICD-9-CM codes: 430–438), head injury (850–854, 95901), diabetes mellitus (250), hyperlipidemia (272), heart failure (428), hypertension (401–405), coronary artery disease (410–414), chronic obstructive pulmonary disease (490–496), asthma (493), ischemic stroke (433, 434, 436, 437.1), malignancy (140–208), chronic kidney disease (580–587), arrhythmia (427, 785.0, 785.1), Parkinson’s disease (332), depression (296.2, 296.3; 300.4, 309.0, 309.1, 311), anxiety (300.0, 300.2, 300.3, 308.3, and 309.81), epilepsy (345), alcoholism (303, 305.0, V113) within 1 year before their index admission as previous studies [16–24]. In addition, information about several possible confounders—length of ICU stay, length of hospital stay, number of ICU readmissions, and duration of MV use—were collected.

### Endpoint

The primary endpoint of the study was newly diagnosed dementia (ICD-9-CM codes 290.0–290.9, 331.0–331.2, and 294.1–294.11) as previously described [19]. Patients were followed from January 1, 1999, until the earliest onset of one of two occurrences, whichever came first: newly diagnosed dementia or end of the study, December 31, 2013. The demographic and clinical characteristics of age, gender, length of ICU and hospital stays, ICU readmission, duration of MV use, and comorbidities were used to estimate the risk of dementia.

### Statistical analysis

The mean (standard deviation) and frequency (percentage) are presented as continuous variables and discrete variables, respectively. Differences in baseline characteristics between groups were evaluated using Pearson’s χ^2^ test for categorical variables. Kaplan-Meier curves were used to plot the cumulative incidence rates of dementia among mechanically ventilated patients, and a log-rank test was used to compare the differences in risks between subgroups. In addition, Cox proportional hazard regression was used to calculate the risk of dementia among ICU patients with MV during the follow-up period. To examine the potential risk factors of dementia, two selection models were used. Model one includes all potential risk factors listed in Table 1 and model two is a Collett’s model [20], which used a significance level of 0.10 for the univariate screening and a stay and entry criterion of 0.05. The Collect’s model selection approach was based on the assumption that treated all variables as the same effect, so the related risk factors could be found in the final model. SAS 9.4 (SAS Institute, Cary, NC, USA) was used for all statistical analyses. Significance was set at *P* < 0.05 (two-sided). Kaplan-Meier curves were plotted using Stata 12 (Stata Corp., College Station, TX, USA).

**Table 1:** Characteristics of mechanically ventilated patients in the intensive care unit (ICU).

Characteristic	Overall	Dementia	Non-Dementia	*p*
	(n = 18,033)	(n = 1387)	(n = 16,646)	
**Age at entry**, years: mean ± SD	70.0 ± 10.9	74.4 ± 9.6	69.6 ± 10.9	< 0.0001
50–64	6237 (34.59)	235 (16.94)	6002 (36.06)	< 0.0001
65–74	5425 (30.08)	442 (31.87)	4983 (29.94)	
75–84	4808 (26.66)	528 (38.07)	4280 (25.71)	
≥ 85	1563 (8.67)	182 (13.12)	1381 (8.3)	
**Gender**				
Female	7320 (40.59)	664 (47.87)	6656 (39.99)	< 0.0001
Male	10713 (59.41)	723 (52.13)	9990 (60.01)	
**Pre-existing risk**				
Hospital length of stay, days: median (IQR)	16 (9 to 26)	19 (13 to 29)	16 (9 to 26)	0.0084
ICU length of stay, days: median (IQR)	5 (2 to 12)	7 (3 to 14)	5 (2 to 12)	0.0864
Duration of ventilated days, days: median (IQR)	3 (2 to 10)	4 (2 to 11)	3 (2 to 10)	0.2342
1–2 days	7780 (43.14)	517 (37.27)	7263 (43.63)	< 0.0001
3–10 days	6071 (33.67)	501 (36.12)	5570 (33.46)	
> 10 days	4182 (23.19)	369 (26.6)	3813 (22.91)	
**Readmission of ICU**				
No	302 (1.67)	5 (0.36)	297 (1.78)	< 0.0001
Yes	17731 (98.33)	1382 (99.64)	16349 (98.22)	
No. of readmission of ICU: median (IQR)	2 (1 to 3)	2 (2 to 3)	2 (1 to 3)	0.8258
1	5130 (28.45)	316 (22.78)	4814 (28.92)	< 0.0001
2	7964 (44.16)	691 (49.82)	7273 (43.69)	
≥ 3	4637 (25.71)	375 (27.04)	4262 (25.6)	
**Comorbidities**				
Hypertension	7483 (41.50)	687 (49.53)	6796 (40.83)	< 0.0001
Diabetes mellitus	4445 (24.65)	304 (21.92)	4141 (24.88)	0.014
Chronic obstructive pulmonary disease	2661 (14.76)	216 (15.57)	2445 (14.69)	0.372
Coronary artery disease	2321 (12.87)	200 (14.42)	2121 (12.74)	0.073
Hyperlipidemia	1777 (9.85)	148 (10.67)	1629 (9.79)	0.2884
Arrhythmia	1246 (6.91)	116 (8.36)	1130 (6.79)	0.0263
Chronic kidney disease	1179 (6.54)	68 (4.90)	1111 (6.67)	0.0103
Heart failure	907 (5.03)	57 (4.11)	850 (5.11)	0.1027
Asthma	902 (5.00)	65 (4.69)	837 (5.03)	0.5747
Stroke	764 (4.24)	90 (6.49)	674 (4.05)	< 0.0001
Ischemic stroke	718 (3.98)	85 (6.13)	633 (3.80)	< 0.0001
Anxiety	629 (3.49)	63 (4.54)	566 (3.40)	0.0259
Depression	436 (2.42)	73 (5.26)	363 (2.18)	< 0.0001
Malignancy	396 (2.20)	6 (0.43)	390 (2.34)	< 0.0001
Parkinson’s disease	357 (1.98)	95 (6.85)	262 (1.57)	< 0.0001
Head injury	179 (0.99)	21 (1.51)	158 (0.95)	0.0415
Epilepsy	111 (0.62)	17 (1.23)	94 (0.56)	0.0025
Alcoholism	41 (0.23)	3 (0.22)	38 (0.23)	1
Hemorrhagic stroke	33 (0.18)	2 (0.14)	31 (0.19)	1
**Follow-up time**, years: median (IQR)	8.1 (4.1–11.5)	3.2 (1.3–6.3)	8.4 (4.6–11.6)	< 0.0001
**Time to dementia**, years: median (IQR)	–	3.2 (1.3–6.3)	–	–
**Time to dementia (within 5 years)**, years: median (IQR)	–	1.8 (0.8–3.2)	–	–

Data are presented as n (%) unless otherwise indicated.

SD = standard deviation, IQR = interquartile range

## Results

**Fig 2:**
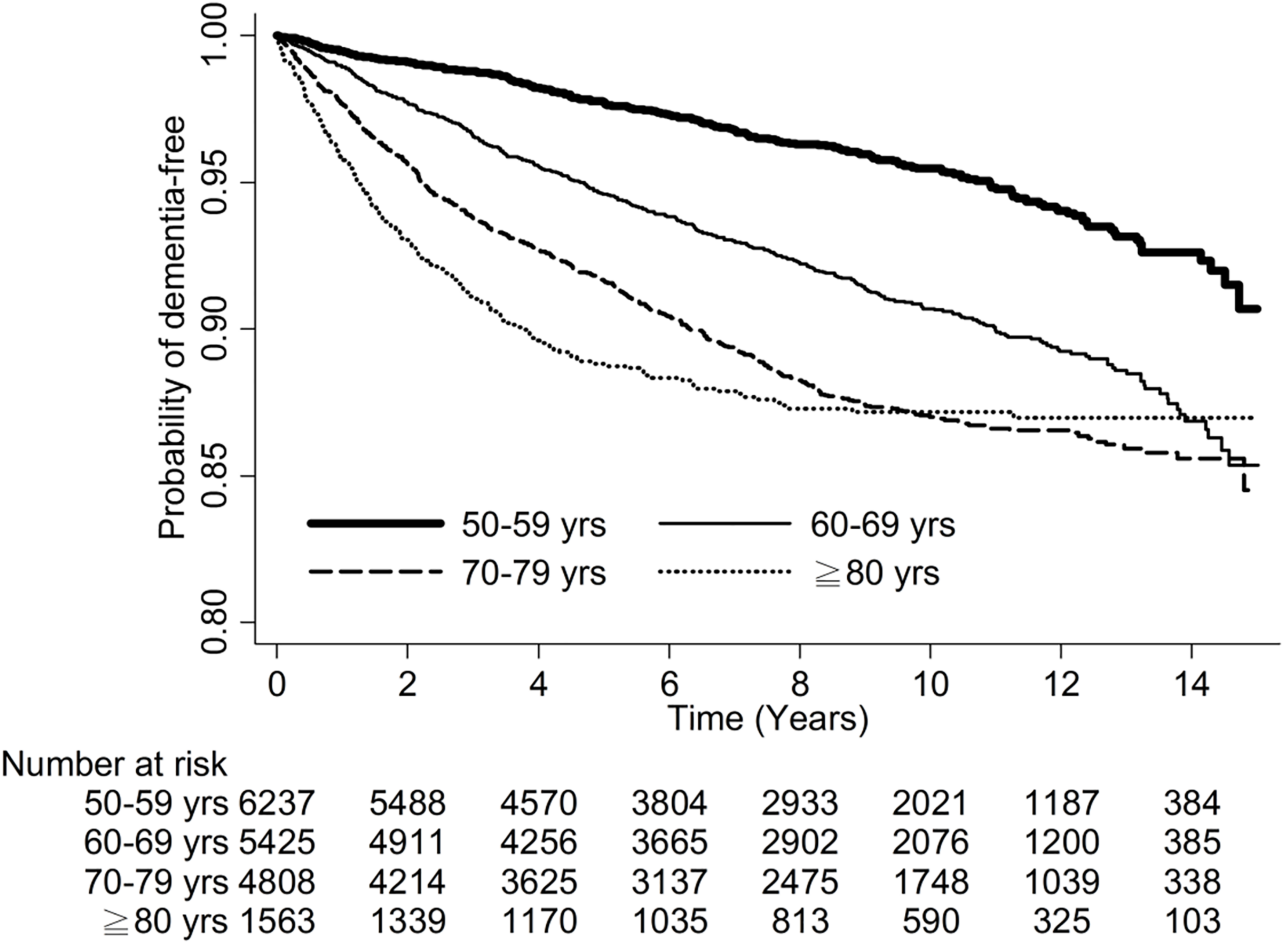
Kaplan-Meier curve shows the probability of being dementia-free after hospital discharge for patients admitted to the intensive care unit and mechanically ventilated.

During the study period, 18,033 patients were enrolled (mean follow-up: 8.1 years) (Table 1, Fig 2). There were 1387 patients with newly diagnosed dementia. The overall incidence of dementia was 9.83 per 1000 patient-years (1387 events for 141,121 person-years), and the mean onset of dementia was 3.2 years from ICU admission and MV (overall), and 1.8 years within the first five years of follow-up. Patients who subsequently developed dementia were more likely to be older, female, have had a longer hospital stay, have been readmitted to the ICU, have had more strokes, hypertension, arrhythmia, anxiety, depression, Parkinson’s disease, head injury, or epilepsy than were patients without dementia.

**Fig 3:**
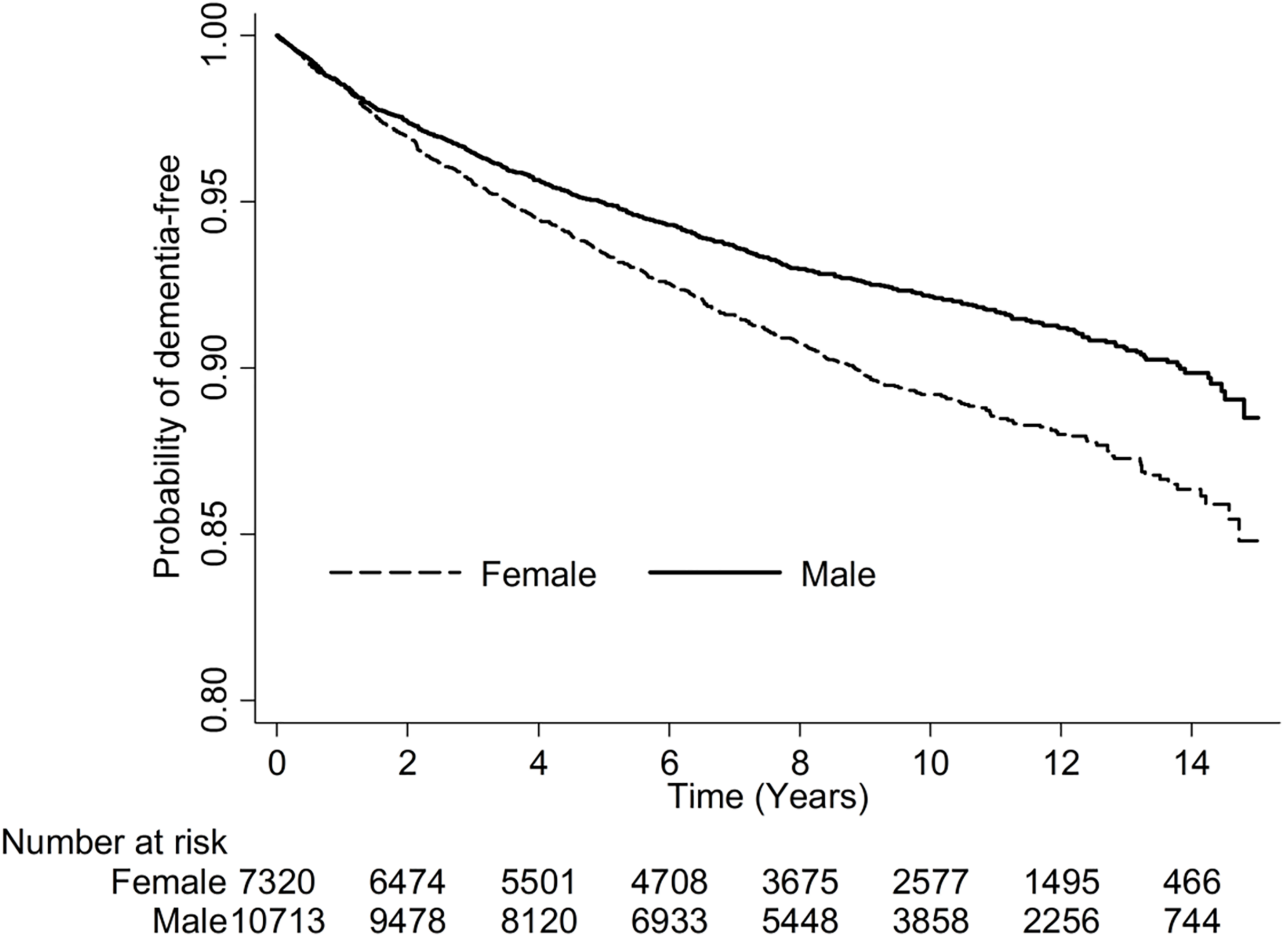
Kaplan-Meier curves show the probability of being dementia-free after hospital discharge for patients admitted to the intensive care unit and mechanically ventilated by age group (A), and by gender group(B) (*p* < 0.0001, log-rank test).

Patients ≥ 85 years old had the highest risk of dementia in Model 1 (HR: 2.68, 95% CI: 2.2–3.27 vs. HR: 2.56, 95% CI: 2.18–3 [75–84 years old] vs. HR: 1.92, 95% CI: 1.63–2.25 [65–74 years old]) and in Model 2 (HR: 2.66, 95% CI: 2.18–3.24 vs. vs. HR: 2.54, 95% CI: 2.17–2.97 [75–84 years old] vs. HR: 1.90, 95% CI: 1.62–2.23 [65–74 years old]) using multivariate analysis (Table 2). Male patients had a lower risk of dementia than did female patients in both models (HR: 0.81, 95% CI: 0.72–0.9 in Model 1; HR: 0.80, 95% CI: 0.72–0.89 in Model 2). Patients with longer ICU stays (> 5 days) had a higher risk of dementia than did patients with ICU stays of ≤ 5 days in both models (HR: 1.20, 95% CI: 1.07–1.35 in Model 1; HR: 1.20, 95% CI: 1.07–1.35 in Model 2). Patients with longer hospital stays (> 14 days) had a higher risk of dementia than did patients with hospital stays of ≤ 14 days in both models (HR: 1.35, 95% CI: 1.2–1.51 in Model 1; HR: 1.34, 95% CI: 1.2–1.51 in Model 2). Patients readmitted to the ICU had a higher risk of dementia than did patients not readmitted to the ICU in both models (HR: 4.76, 95% CI: 1.97–11.49 in Model 1; HR: 4.76, 95% CI: 1.97–11.48 in Model 2). Finally, patients with hypertension, hyperlipidemia, depression, Parkinson’s disease, and epilepsy had a higher risk of dementia than did patients without these comorbidities. In contrast, patients with DM, chronic kidney disease, heart failure, and malignancy had a lower risk of dementia than did patients without these comorbidities. Older patients had a higher incidence rate of dementia than did younger patients, and women had a higher incidence rate than did men (both *p* < 0.0001) (Fig 3).

**Table 2:** Multivariate analysis for the risk of dementia.

Variables	Univariate			Model I: Multivariate*		Model II: Multivariate**	
	HR (95% CI)		*p*	HR (95% CI)	*p*	HR (95% CI)	*p*
**Age**, years: mean ± SD							
50–64	1.00			1.00		1.00	
65–74	2.03 (1.74–2.38)		< 0.0001	1.92 (1.63–2.25)	< 0.0001	1.90 (1.62–2.23)	< 0.0001
75–84	2.82 (2.42–3.29)		< 0.0001	2.56 (2.18–3.00)	< 0.0001	2.54 (2.17–2.97)	< 0.0001
≥ 85	3.02 (2.49–3.66)		< 0.0001	2.68 (2.20–3.27)	< 0.0001	2.66 (2.18–3.24)	< 0.0001
**Gender**							
Female	1.00			1.00		1.00	
Male	0.74 (0.67–0.82)		< 0.0001	0.81 (0.72–0.90)	< 0.0001	0.80 (0.72–0.89)	< 0.0001
**Pre-existing risk**							
ICU length of stay							
≤ 5 days	1.00			1.00		1.00	
> 5 days	1.51 (1.36–1.68)		< 0.0001	1.20 (1.07–1.35)	0.0016	1.20 (1.07–1.35)	0.0018
Hospital length of stay							
≤ 14 days	1.00			1.00		1.00	
> 14 days	1.49 (1.34–1.66)		< 0.0001	1.35 (1.20–1.51)	< 0.0001	1.34 (1.20–1.51)	< 0.0001
Readmission to ICU							
No	1.00			1.00		1.00	
Yes	5.94 (2.47–14.28)		< 0.0001	4.76 (1.97–11.49)	0.0005	4.76 (1.97–11.48)	0.0005
**Comorbidities**							
Hypertension	1.47 (1.33–1.64)		< 0.0001	1.35 (1.21–1.52)	< 0.0001	1.36 (1.22–1.52)	< 0.0001
Diabetes mellitus	0.82 (0.72–0.93)		0.0018	0.70 (0.61–0.80)	< 0.0001	0.71 (0.62–0.81)	< 0.0001
Coronary artery disease	1.17 (1.00–1.35)		0.0462	0.98 (0.83–1.14)	0.7565		
COPD	1.04 (0.90–1.21)		0.5768	0.94 (0.79–1.11)	0.4554		
Hyperlipidemia	1.38 (1.17–1.64)		0.0002	1.45 (1.21–1.73)	< 0.0001	1.46 (1.22–1.74)	< 0.0001
Chronic kidney disease	0.72 (0.57–0.92)		0.0091	0.71 (0.56–0.91)	0.0065	0.71 (0.55–0.91)	0.0059
Arrhythmia	1.30 (1.07–1.57)		0.0075	1.08 (0.89–1.32)	0.4493		
Heart failure	0.82 (0.63–1.07)		0.1391	0.69 (0.52–0.90)	0.0074	0.68 (0.52–0.89)	0.0048
Stroke	1.49 (1.20–1.84)		0.0003	1.56 (0.60–4.04)	0.3601		
Ischemic stroke	1.50 (1.20–1.86)		0.0003	0.75 (0.28–1.98)	0.5617		
Hemorrhagic stroke	0.69 (0.17–2.76)		0.5989	0.40 (0.09–1.78)	0.2274		
Asthma	0.90 (0.70–1.15)		0.4019	0.82 (0.62–1.10)	0.1912		
Anxiety	1.51	(1.17–1.94)	0.0015	1.19 (0.92–1.55)	0.1888		
Depression	2.73 (2.16–3.46)		< 0.0001	2.17 (1.69–2.78)	< 0.0001	2.25 (1.77–2.87)	< 0.0001
Parkinson’s disease	4.19 (3.40–5.16)		< 0.0001	2.84 (2.29–3.53)	< 0.0001	2.88 (2.32–3.57)	< 0.0001
Malignancy	0.18 (0.08–0.40)		< 0.0001	0.21 (0.09–0.46)	0.0001	0.21 (0.09–0.47)	0.0001
Head injury	1.45 (0.94–2.23)		0.0934	1.23 (0.80–1.90)	0.3507		
Epilepsy	2.29 (1.42–3.69)		0.0007	1.94 (1.20–3.16)	0.0071	1.98 (1.22–3.21)	0.0054
Alcoholism	1.15 (0.37–3.56)		0.8126	1.68 (0.54–5.25)	0.3758		

*All variables in the model

**Collett’s Model Selection Approach for the best model fitted to the data.

COPD = chronic obstructive pulmonary disease, ICU = intensive care unit, IQR = interquartile range, HR = hazard ratio, CI = confidence interval, SD = standard deviation

## Discussion

This is the first study that investigates (1) the long term risk of dementia in patients admitted to the ICU and put on MV, and (2) the effects of MV on the risk of dementia of specific groups of critically ill patients. Our most important finding is that of 18,033 patients admitted to the ICU and put on MV, those who survived to hospital discharge for at least 6 months, dementia was newly diagnosed in 1387 (141,121 person-years), and the overall incidence was 9.83 per 1000 person-years over the 8 years of follow-up. The mean onset of dementia was 3.2 years from ICU admission and MV. Guerra et al. [8] reported that the rate of newly diagnosed dementia in 10,348 patients admitted to the ICU was 15.0% (1648 patients), and the incidence was 73.6 per 1000 person-years over a 3-year follow-up (based on a random 2.5% sample of Medicare beneficiaries who received ICU care and survived to hospital discharge). In contrast to Guerra et al. [8] enrolled patients ≥ 66 years old, we enrolled patients ≥ 50 years old, and patients 50–64 years old comprised 35% of our study population. In addition, we enrolled only patients admitted to the ICU and put on MV, and all of our patients were Taiwanese. These differences might partially explain why our findings are different from other studies [4, 8, 9, 11] that enrolled primarily Caucasian patients admitted to the ICU but not put on MV. The present study specifically focused on Taiwanese patients admitted to the ICU and put on MV who survived to hospital discharge and then underwent a long-term follow-up.

Second, we found the subsequent development of dementia among MV patients admitted to the ICU was positively associated with age and gender: older patients and women had higher risks of dementia. This is consistent with recent study with the findings of Guerra et al [9]. Taken together, the above findings indicate that clinicians should keep in mind the development of dementia, especially in elderly female patients who survived after undergoing MV in the ICU.

Third, several hospital factors—length of ICU stay, ICU readmission, and length of hospital stay—were independently associated with the risk of dementia in two multivariate models in this study. Again, this is consistent with the findings of Guerra et al. [9] that long ICU and hospital stays increase risk of dementia. Moreover, Ehlenbach et al. [4] reported a similar effect of hospitalization itself on the development of dementia in patients > 65 years old. All of these findings should emphasize the significant effect of ICU and hospital stays on the development of dementia.

Our study has some strength. It is large, population-based—which allows it to avoid the effect of referral bias—and has a long-term follow-up analysis of the association between the effect of ICU admission plus MV and the development of dementia. However, we had some unusual findings regarding the negative association between dementia and several well-known risk factors including DM, CKD and malignancy among this specific population. These findings are contrary to previous studies [15, 16]. Despite the differences may be due to the different study subjects and design, further investigations is needed to clarify these issues. Finally, to choose the major risk factors in this study, we used Collett’s model selection approach. Furthermore, these established prognostic baseline clinical factors can be used to assess the risk of developing dementia after an ICU stay.

Our study also has some limitations. First, although our study relies on large nationwide administrative databases, we might not have eliminated all residual confounders. Moreover, the primary reasons for admitting these patients with MV and the weaning status after ICU discharge are unclear, as are additional details about the severity of their ARF. In addition, the data about baseline pre-ICU cognitive function of patients is lack. Preadmission mild cognitive dysfunction in the early stage of dementia is known to increase risk of ICU admission; however, they may have gone undiagnosed. Finally, the enrolled patients were selected from a heterogeneous general population, which might prevent us from generalizing our conclusions. However, given the large magnitude of the observed effects in this study, these limitations should have only a minimal effect on our findings.

In conclusion, the long-term risks of subsequent dementia in patients admitted to the ICU, put on MV, and then survive to hospital discharge increase with age and are higher in women than in men. Additionally, longer ICU hospital stays and more ICU readmissions are significantly associated with developing dementia in this specific group.

## Abbreviations

ARF: acute respiratory failure
ICU: intensive care unit
MV: mechanical ventilation
NHIRD: National Health Insurance Research Database
